# Estimating the synthetic accessibility of molecules with building block and reaction-aware SAScore

**DOI:** 10.1186/s13321-024-00879-0

**Published:** 2024-07-23

**Authors:** Shuan Chen, Yousung Jung

**Affiliations:** 1 Department of Chemical and Biological Engineering, Seoul National University, 1 Gwanak-ro, Gwanak-gu, Seoul, 08826 South Korea; 2 Institute of Chemical Processes, Seoul National University, 1 Gwanak-ro, Gwanak-gu, Seoul, 08826 South Korea; 3 Institute of Engineering Research, Seoul National University, 1 Gwanak-ro, Gwanak-gu, Seoul, 08826 South Korea

**Keywords:** Synthetic accessibility, Synthesis planning, Chemical reactivity, Building-block accessibility

## Abstract

Synthetic accessibility prediction is a task to estimate how easily a given molecule might be synthesizable in the laboratory, playing a crucial role in computer-aided molecular design. Although synthesis planning programs can determine synthesis routes, their slow processing times make them impractical for large-scale molecule screening. On the other hand, existing rapid synthesis accessibility estimation methods offer speed but typically lack integration with actual synthesis routes and building block information. In this work, we introduce BR-SAScore, an enhanced version of SAScore that integrates the available building block information (B) and reaction knowledge (R) from synthesis planning programs into the scoring process. In particular, we differentiate fragments inherent in building blocks and fragments to be derived from synthesis (reactions) when scoring synthetic accessibility. Compared to existing methods, our experimental findings demonstrate that BR-SAScore offers more accurate and precise identification of a molecule's synthetic accessibility by the synthesis planning program with a fast calculation time. Moreover, we illustrate how BR-SAScore provides chemically interpretable results, aligning with the capability of the synthesis planning program embedded with the same reaction knowledge and available building blocks.

**Scientific contribution**

We introduce BR-SAScore, an extension of SAScore, to estimate the synthetic accessibility of molecules by leveraging known building-block and reactivity information. In our experiments, BR-SAScore shows superior prediction performance on predicting molecule synthetic accessibility compared to previous methods, including SAScore and deep-learning models, while requiring significantly less computation time. In addition, we show that BR-SAScore is able to precisely identify the chemical fragment contributing to the synthetic infeasibility, holding great potential for future molecule synthesizability optimization.

**Supplementary Information:**

The online version contains supplementary material available at 10.1186/s13321-024-00879-0.

## Introduction

In recent years, there has been a surge in the development of generative models aimed at proposing potential drug or functional material candidates [1–3]. However, despite the promising chemical or biological properties attributed to these generated molecules, the challenge lies in translating these virtual designs into real synthesis, posing a significant bottleneck [4, 5]. Although the emergence of synthesizability-ensured molecule design has posed a promising solution to design more synthesizable molecules, most of the other existing inverse design algorithms still suffer from this synthesizability issue [6–9]. Fortunately, with the advancement of computer-aided synthesis planning (CASP) algorithms [10, 11], scientists now can access the synthesis pathways of virtually designed molecules without the need for manual retrosynthesis analysis, thus expediting the screening process. To further streamline this process, several algorithms have emerged to predict the synthetic accessibility of organic molecules directly from their structural features, bypassing the need for running time-consuming synthesis planning programs [12–14]. For instance, Thakkar et al. [13] proposed a machine learning (ML)-based scoring function called RAScore to rapidly estimate whether the synthesis route of a given molecule can be successfully planned by the synthesis planning program AizynthFinder [15] or not. The time of running a synthesis planning program for 200,000 molecules sampled from the ChEMBL databse [16] was significantly reduced from 239 days to 79 min by a using RAScore. Wang et al. [14] proposed a language-based ML model to predict whether the synthesis route of a given molecule can be found by Retro* [17], another synthesis planning program. Extra filter such as generic cyclic feature score (GCF) [18] was found useful for filtering out exotic associations that are unlikely to be synthesized or appear in the real chemical space.

Despite these advancements, the ML-based methods trained by the molecules labeled by synthesis planning program cannot capture the full picture of the synthesis planning capability of the targeted program, since the labeled examples are unlikely to cover all the learned reactions and building blocks available in the program. Moreorver, the computation time of applying ML-based models is often much longer than the rule-based methods. For example, as shown in the results section, the computation time of RAScore [13] is more than 300 times of that of SAScore [19]. Therefore, designing a much faster scoring function that can sufficiently fully capture the capability of synthesis planning program is needed for practical synthesis accessibility estimation.

In this paper, we introduce Building block and Reaction-aware SAScore (BR-SAScore), a novel approach that rapidly estimates the synthetic accessibility of a molecule enhanced by the knowledge of available building blocks (B) and reaction (R) based on the rule-based method SAScore [19]. Unlike previous ML-based models, which learns from the examples labeled by synthesis planning program, BR-SAScore analyzes molecule fragments to directly represent building block and reaction knowledge of the synthesis planning program of interest. Specifically, we decouple fragmentScore in SAScore into building-block fragment score (BScore) and reaction-driven fragment score (RScore) to explicitly consider synthesis knowledge and building blocks accessibility from the reaction dataset and building blocks, respectively. Our proposed RB-SAScore demonstrates superior accuracy and precision in synthetic accessibility estimation, coupled with fast calculation speeds. Additionally, its chemically intuitive design facilitates intuitive interpretability, shedding light on the specific molecular features contributing to synthesis infeasibility. We anticipate that the development of RB-SAScore will significantly enhance the synthetic accessibility estimation for virtually designed molecules.

## Materials and methods

### Dataset

To demonstrate the practical performance of BR-SAScore across various domains, we selected three distinct test sets previously utilized for evaluating other methods. The first test set (TS1) was compiled by Voršilák et al. [20], comprising 3,581 molecules sampled from the ZINC-15 database [21] and an equal number from the GDB-17 database [22]. The second test set (TS2), collected by Thakkar et al. [13], consisted of 30,348 molecules sampled from ChEMBL [16], GDBChEMBL [23], and GDBMedChem [24]. Lastly, the third test set (TS3), gathered by Yu et al. [25], comprised 1,800 structural complex molecules sourced from previous works of synthetic accessibility and molecular complexity analysis [19, 20, 26–30].

The labeling of molecules as either easy-to-synthesize (ES) or hard-to-synthesize (HS) differs across these test sets. In TS1, labels are defined based on the source database (ZINC-15 labeled as ES, GDB-17 as HS). Conversely, in TS2 and TS3, labels are determined by whether the synthesis route of the target molecules can be resolved by synthesis planning program Retro* [17]. To ensure label consistency, we standardized the datasets by sampling an equal number of molecules (900 ES and 900 HS) from each test set and relabeling them by employing Retro* for all 5400 molecules to ascertain their synthesis routes. Following established methodologies, a molecule is labeled as ES if its synthesis route can be identified within 10 reaction steps using Retro*, otherwise it is labeled HS. The statistical details of the relabeled datasets are presented in Table 1, and the hyperparameters of implementing Retro* in this paper can be found in Table S1.

**Table 1:** The statistics of 3 test sets labeled by Retro* in this paper

Test set	Source of molecules	# ES molecules	# HS molecules
TS1	ZINC-15 [21] and GDB-17 [22]	745	1055
TS2	ChEMBL [16], GDBChEMBL [23], and GDBMedChem [24]	858	942
TS3	Various sources [19, 20, 26–30]	810	990

### SAScore: a brief review

Our method, RB-SAScore, is based on the SAScore [19], a widely accepted and well-performing synthetic accessibility metric [5, 31]. SAScore integrates both local and global structural molecular features, with local structure represented by molecule fragments (fragmentScore) and global structure represented by structure complexity (complexityPenalty):

1 $$SAScore=fragmentScore-complexityPenalty$$

The fragment score is derived from the popularity of each molecular fragment, encoded as Extended-Connectivity Fingerprints [32] (ECFPs), among a set of previously synthesized molecules. The rationale is that fragments appearing more frequently across diverse molecules are more likely to be synthesized, while rare fragments receive negative scores. By fragmenting 934,046 molecules from the PubChem databasese [28], the score of each fragment is computed, with common fragments receiving higher scores and rare ones assigned negative scores. These fragment scores are then averaged to represent the overall local feature of a given molecule.

2 $$fragmentScore=\frac{\sum_{k=i}^{n}{Score}_{i}}{n}$$

On the other hand, global features such as the number of atoms and stereocenters in the molecule are captured by the complexity penalty term. Specifically, the complexity penalty comprises four commonly considered features in synthesis accessibility: size complexity (number of atoms), stereo complexity (number of stereocenters), ring complexity (number of bridgehead and spiro atoms), and macrocycle complexity (number of rings with size > 8). Mathematically, they are calculated as follows:

3 $$complexityPenalty=SizeComplexity+StereoComplexity+RingComplexity+MacrocycleComplexity$$

where

4 $$SizeComplexity={{n}_{Atoms}}^{1.005}-{n}_{Atoms}$$

5 $$StereoComplexity=log({n}_{ChiralCenter}+1)$$

6 $$RingComplexity=log\left({n}_{Bridgehead}+1\right)+log\left({n}_{SpiroAtoms}+1\right)$$

7 $$MacrocycleComplexity=log({n}_{MacroCycle}+1)$$

Finally, the calculated score from Eq. 1 is multiplied by -1 and scaled between 1 and 10, where molecules with higher SAScore are predicted to be more difficult to synthesize, while those with lower SAScore are predicted to be easier to synthesize.

The distribution of structural complexity for the ES and HS molecules in the three test sets is depicted in Figure S1. Overall, the size penalty and stereo penalty of molecules in TS3 are higher than those in TS1 and TS2, indicating more complex molecular structures in TS3 compared to TS1 and TS2. Additionally, the penalty difference between ES molecules and HS molecules increases progressively from TS1 to TS3.

### Building-block reaction-driven and fragments

While the original SAScore offers valuable intuition and applicability across a wide range of molecules, it lacks consideration for individual chemical knowledge and building block accessibility. Simply because a molecule has been previously synthesized and cataloged in the PubChem database may not guarantee its synthesizability since the actual reaction routes are not considered. Moreover, SAScore may exhibit over-pessimism towards synthesizability for molecules containing chemical fragments commonly found in building blocks but absent in the PubChem database, potentially due to biased molecule sampling.

To bridge this gap between SAScore and these additional considerations (reactions and building blocks), we propose substituting the fragmentScore in Eq. 1 with BR-fragmentScore, which encompasses fragments explicitly representing the learned reaction and available building blocks embedded in the synthesis planning program:

8 $$BR-SAScore=BR-fragmentScore-complexityPenalty$$

**Fig. 1:**
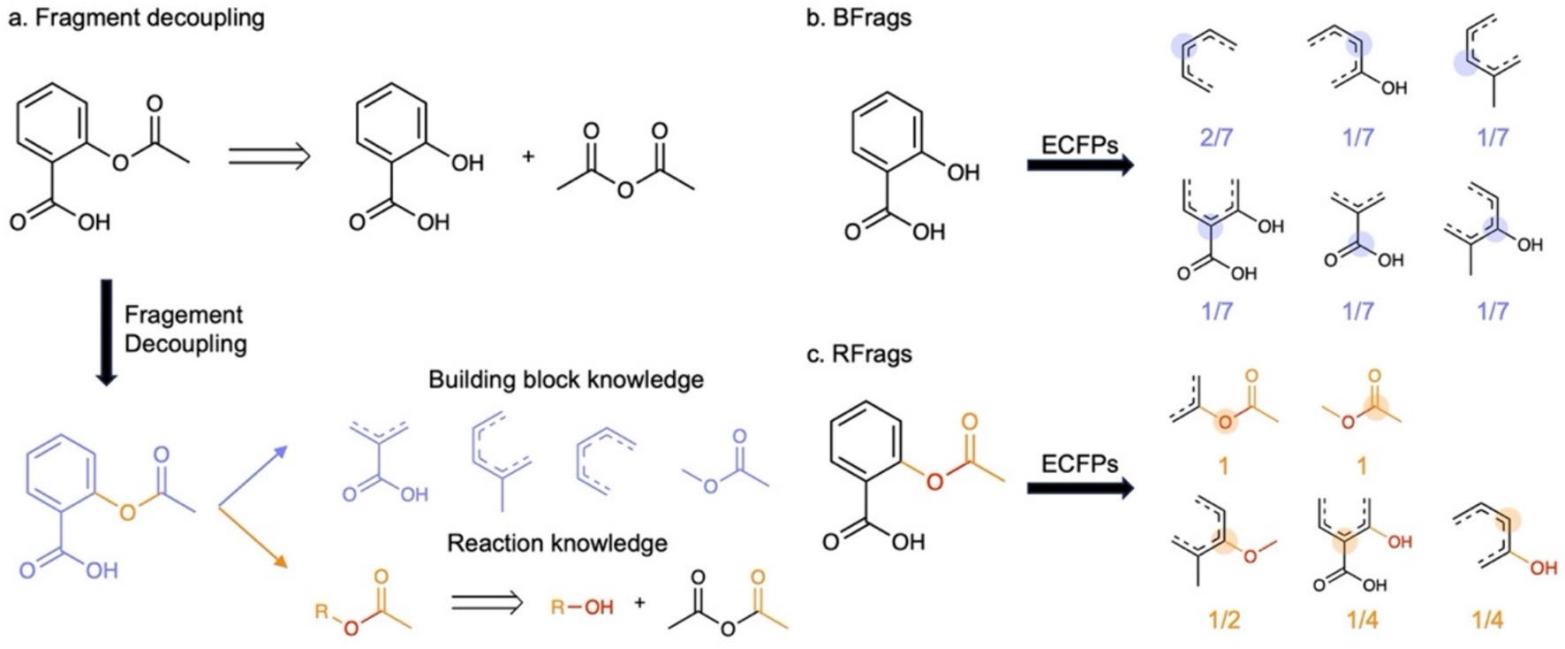
The workflow of calculating the BR-fragmentScore. **a** The fragments of a given molecule can be decomposed to the fragments from buildings block (blue) and reaction (orange) knowledge. **b** The buildings block knowledge is represented by the fragments existing in the known building blocks, denoted as BFrags. **b** The reaction knowledge is represented by the fragments participating in the known reactions, denoted as RFrags

Intuitively, a molecule’s fragments can be categorized into two types: those inherent in the building blocks (building block fragments, or BFrags) and those formed after chemical reactions (reaction-driven fragments, or RFrags). For instance, consider Aspirin synthesis, where the ester group originates from the reaction and the remaining fragments from the building blocks (Fig. 1a). By deriving BFrags from building blocks and RFrags from reaction datasets, we assemble a set of fragments applicable for molecule construction. We assume that if all fragments in a given molecule match the popular fragments in the derived set, either BFrags or RFrags, the molecule is likely to be synthetically accessible.

To calculate BR-fragmentScore, we derive BScore and RScore from BFrags and RFrags akin to the derivation of fragmentScore from chemical fragments in SAScore with a few modifications. For BScore, we extract the Extended-Connectivity Fingerprints [32] with radius equals 2 (ECFP4) of each atom in accessible building blocks, and the popularity of fragments within the entire building block set is calculated (Fig. 1b). Recognizing that chemical fragments in larger building blocks are less versatile for synthesis, we normalize fragment counts by the total number of fragments extracted from the molecule. For RScore, we capture reaction centers using a reaction template extraction algorithm based on atom-to-atom mapping [33–35]. By extracting the ECFP4 of atoms in the reaction center and the neighboring atoms in the reaction product, RFrags are extracted for all recorded reactions (Fig. 1c). Additionally, we extract fragments from atoms two-hop away from the reaction center to enhance the description of the reaction environment. Because distant fragments would have less impact on the reaction, we weigh the fragment counts by ${2}^{-d}$, where $d$ is the shortest distance from the atom to the reaction center. To prevent the biases caused by frequently appeared small fragments, we exclude the chemical fragments with radius equal 0 and 1 in ECFP4.

Next, we transform the counts of each BFrag and RFrag collected from the reaction dataset and building blocks into BScore and RScore by applying the logarithmic function after dividing the count of the fragment by 0.1% of the total number of fragments derived from the dataset (${N}_{B}$ for building blocks, ${N}_{R}$ for reaction dataset), as shown in Eq. 9 and 10. To reduce the bias from the extremely rare fragments, fragment counts no more than 1 are excluded. Subsequently, the RScores and BScores are scaled between − 3 and 3.

9 $${BScore}_{i}=\text{log}\left(\frac{{count}_{i}}{0.001{N}_{B}}\right)$$

10 $${RScore}_{i}=\text{log}\left(\frac{{count}_{i}}{0.001{N}_{R}}\right)$$

If a fragment $i$ is found in the recorded BFrags and RFrags, the fragment's score is determined by the higher score value; if the fragment $i$ is not found in the recorded BFrags or RFrags, the score of the fragment is set to − 6.

11 $${Score}_{i}=\text{max}\left({BScore}_{i}, {RScore}_{i}\right)$$

For a given molecule, BR-fragmentScore is calculated by averaging the non-positive terms of ${Score}_{i}$ after enumerating all n fragments extracted from the given molecule.

12 $$BR-fragmentScore=\frac{\sum_{k=i}^{n}{Score}_{i}}{n} if {Score}_{i}<0$$

Finally, the BR-SAScore is calculated using Eq. 8 along with the complexityPenalty term defined in SAScore, and the score is scaled between 1 and 10 following the scale of the original SAScore:

13 $${\text{BR}-\text{SAScore}}_{normalized}=10-9(\frac{\text{BR}-\text{SAScore}-(-6-\text{PenaltyBuffer})}{0-(-6-\text{PenaltyBuffer})})$$

**Fig. 2:**
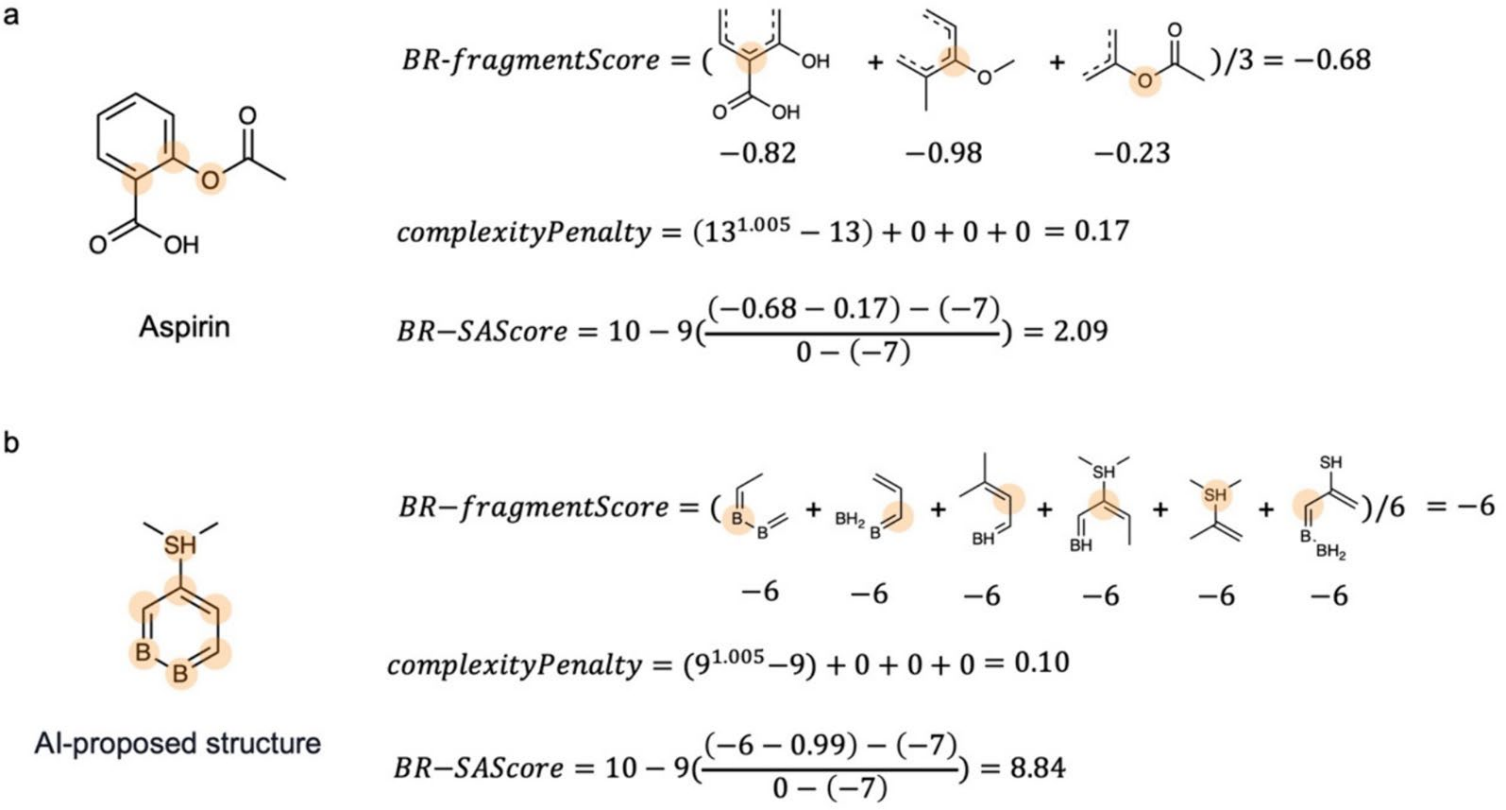
The examples of calculating the BR-SAScore for (**a**) Aspirin and (**b**) an AI-proposed structure shown in Gao et al.[5]. While Aspirin has low BR-SAScore (easy-to-synthesize), the AI-proposed structure has high BR-SAScore (hard-to-synthesize) due to the rare fragments in the molecule. The chemical fragments in the examples with non-zero BR-fragmentScore are highlighed in orange

The best BR-SAScore is 1 if there is no any rare fragment or complex fragment in the molecule, and the worst BR-SAScore is 10 if all the fragments in the molecule do not appear in the reaction database or building blocks (${Score}_{i}=-6$) and the molecule has high structural complexity. Thus, the BR-SAScore is normalized by the maximum value $0$ and minimum value $-6-\text{PenaltyBuffer}$, where $\text{PenaltyBuffer}$ is the additional buffer for differentiating molecules with rare fragments having different structural complexity. The default value of $\text{PenaltyBuffer}$ is set to 1 in this paper. Examples of calculating the BR-SAScore for Aspirin and an AI-proposed molecular structure showed in Gao et al. [5] are provided in Fig. 2.

In this study, our focus lies on the Retro* synthesis program [17], a synthesis planning algorithm that leverages approximately 1 million reactions from the USPTO reaction dataset [36] and 231 million commercially available building blocks cataloged in eMolecules (https://downloads.emolecules.com/free). From these datasets, we calculated scores for a total of 331,332 fragments to estimate the BR-fragmentScore. It's worth noting that our method can be readily customized to other synthesis planning program by accessing their reaction datasets and building blocks. The top-10 BFrags and RFrags with highest scores can be found in Figure S2.

## Results and discussion

### Main results

In this article, we conduct a comparative analysis of BR-SAScore with 6 existing methods, including the likeness-based (SAScore [19] and CLScore [37]) and learning-based methods (SYBA [20], RAScore [13], GASA [25], and DeepSA [14]) ones. Likeness-based methods asseess the synthetic accessibility by estimating the likeness of the given molecules with moelcules collected from the known databases, while learning-based methods typically train a machine learning model to distinguished positive (HS) and negative (HS) molecules. SAScore and CLScore estimate the likeness between the given molecule and the molecules in the databases (PubChem and ChEMBL databases, respectively). SYBA learns synthetic accessibility via Bayesian optimization, while RAScore, GASA, and DeepSA employ artificial neural networks, including forward neural networks, graph attention neural networks, and fine-tuning of pretrained language models, respectively.

**Fig. 3:**
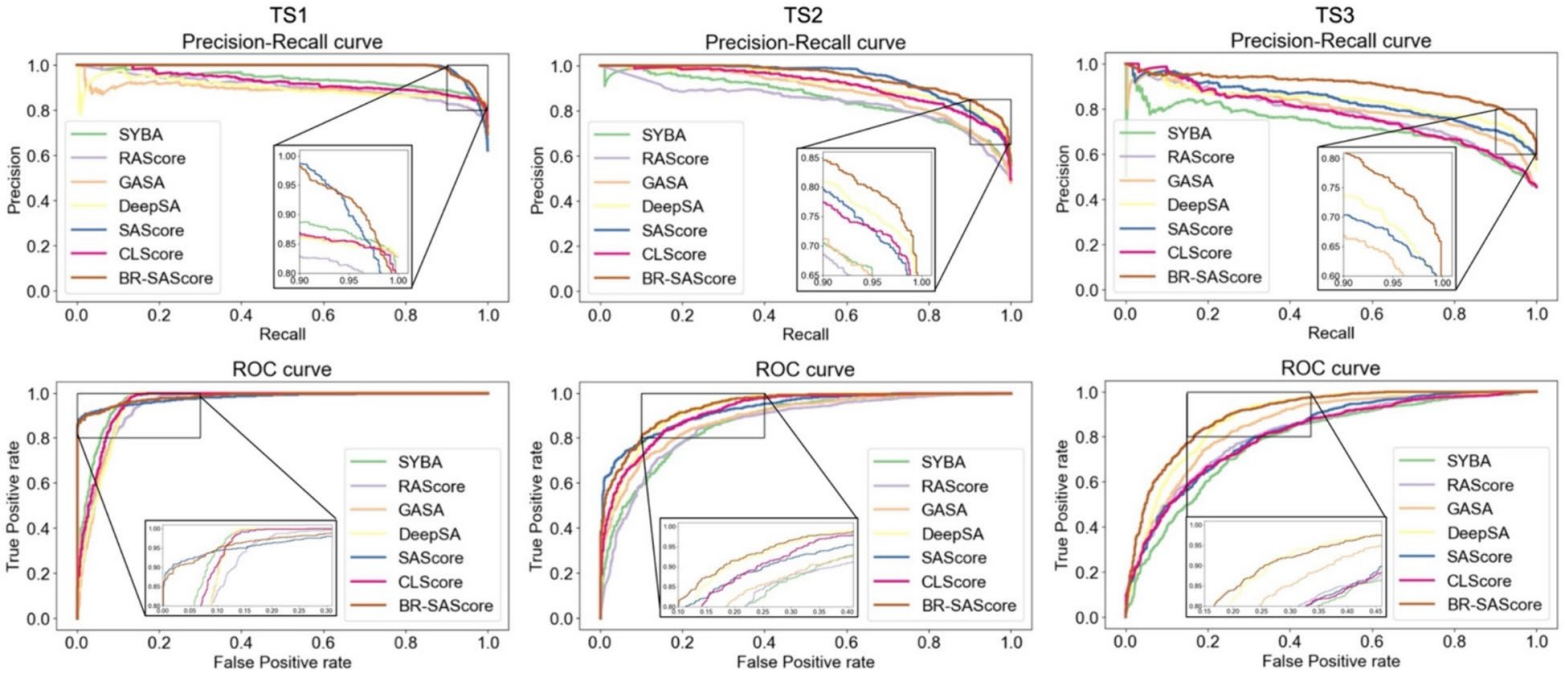
Precision-recall and ROC curves for synthetic accessibility prediction on three test sets using BR-SAScore, compared to six existing methods. Amplified views highlight precision in high-recall areas of the precision-recall curves and performance at low false positive rates in the ROC curves

To evaluate the performance of each method, we present the precision-recall curves and ROC curves of BR-SAScore, tested on three test sets (each comprising 1,800 molecules), compared with 6 existing methods in Fig. 3. Overall, BR-SAScore demonstrates higher precision and true positive rates at nearly all recall and false positive rate values. Specifically, BR-SAScore shows similar curves to SAScore on TS1 and TS2 but exhibits significantly higher precision (~ 0.1) at high recall and lower false positive rate (~ 0.15) at high true positive rates on TS3. These results clearly demonstrate the advantage of incorporating building block and reaction knowledge in BR-SAScore, which enhances the capability of differentiating the synthetic accessibility of complex molecules. Among the other two likeness-based methods, SAScore outperforms CLScore in both precision-recall and ROC curves across all three test sets. When comparing the learning-based methods, SYBA performs best on TS1, whereas DeepSA performs best on TS2 and TS3.

The area under the curves (AUCs) for each method across the three test sets shown in Fig. 3 are calculated in Table 2. Considering the practical applicability for large-scale prediction, we include the computational speed (on CPU) in the same table for comparison. Consistent with the conclusions drawn from Fig. 3, BR-SAScore exhibits the best PR-AUC and ROC-AUC across all test sets, while SAScore and DeepSA show the second-best performance on different test sets and metrics. Although the speed of BR-SAScore is slightly slower (by 7.7%) than SAScore, it is significantly faster (over 40 times) than the encoder of DeepSA. Considering both prediction performance and computational time, BR-SAScore stands out among the previously best methods.

**Table 2:** The results of synthesizability prediction on 3 different test set by 6 different prediction methods

Category	Method	PR-AUC			ROC-AUC			Speed (ms/mol)
		TS1	TS2	TS3	TS1	TS2	TS3	
Learning-based	SYBA [20]	0.939	0.855	0.722	0.968	0.872	0.784	**0.28**
	RAScore [13]	0.905	0.835	0.776	0.948	0.860	0.841	123
	GASA [25]	0.890	0.886	0.801	0.951	0.890	0.851	307
	DeepSA [14]	0.899	0.919	0.832	0.951	0.931	0.886	17.2*
Likeness-based	SAScore [19]	0.988	0.942	0.833	0.980	0.929	0.818	0.39
	CLScore [37]	0.927	0.912	0.776	0.960	0.921	0.808	8.97
	BR-SAScore (this work)	**0.990**	**0.947**	**0.900**	**0.984**	**0.942**	**0.900**	0.42

The best values are highlighted in font bond and the second-best values are underlined. The prediction speed of each method following instructions provided by publicly available source code on GitHub

^*^Prediction speed of DeepSA estimated by running its encoder [38] due to the failed implementation of their provided GitHub scripts

The raw prediction scores of each method can be found in Figure S3, and the ablation study using only BFrags and RFrags in Eq. 11, or using different complexity buffer in Eq. 13 are shown in Figure S4-S6 and Table S2. The value of complexity buffer used in Eq. 11 changes the score distribution but does not change the precision-reall and ROC curves. While BR-SAScore performs well when using only BFrags or only RFrags, utilizing both features consistently achieves the best PR-AUC and ROC-AUC across all test sets.

### Prediction on complex molecules

**Fig. 4:**
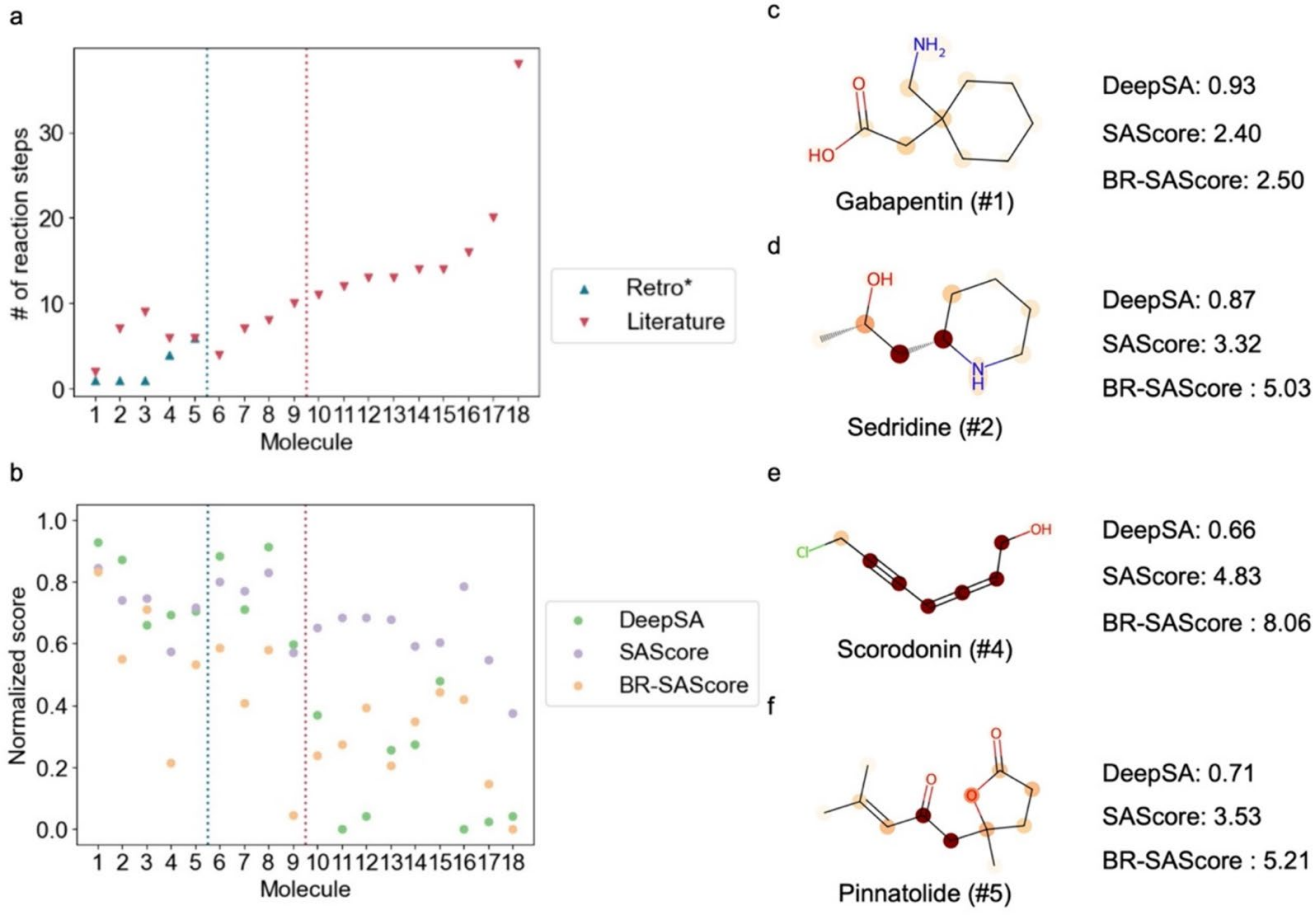
The results of synthesis accessibility estimation on 18 complex molecules collected by Wang et al. [14]. **a** The number of synthesis steps reported in the literature and planned by Retro* [17] for the sampled molecules. Molecules did not solved by Retro* are not shown in the figures. **b** The scores estimated by DeepSA, SAScore, and BR-SAScore on the tested molecules. The blue and red verticle lines in panel a and b are the cutoff lines of molecules being solved by Retro* and the molecules being synthesized within 10 steps reported in literatures. **c**–**f** The predicted scores from DeepSA, SAScore, and BR-SAScore for four of the molecules solved bt Retro*. The negatively contributing atoms (center of fragments) in each molecule given by BR-SAScores. Atoms highlighted with darker color contribute more negative R^2^fragmentScore to the BR-SAScore

To qualitatively analyze the predicted synthetic accessibility of BR-SAScore, we performed an additional test and analysis on 18 complex molecules collected by Wang et al. [14] Since BR-SAScore is designed to reflect the synthesis capability of Retro* [17], we run Retro* to plan the synthesis routes for each molecule to define the synthetic accessibility under Retro* capability. The synthesis planning results for the 18 molecules are shown in Fig. 4a, where the red vertical lines indicate the cutoff lines for successfully planned molecules by Retro* and the molecules synthesized within 10 steps as reported in the literature. Notably, among the 18 examined molecules, only five could be successfully planned by Retro* within 10 steps due to its limited predictive capability. The full list of the synthesis steps predicted by Retro* and those reported in the literature for the 18 tested molecules is provided in Table S4.

Next, we present the results of BR-SAScore predictions on these 18 complex molecules in Fig. 4b. To better compare the results of BR-SAScore with other methods and ensure good visibility, we only show the two best-performing methods, DeepSA and SAScore, and normalize the scores of BR-SAScore and SAScore between 0 (ES) and 1 (HS) in Fig. 4b. The full prediction results by the six existing methods are provided in Figure S7. While the normalized SAScore assigns a high score (> 0.5) to 17 of the tested molecules, DeepSA aligns well with reported reaction steps from the literature but often overestimates the synthetic accessibility for molecules not successfully planned by Retro* (the 6–9th molecules). In contrast, except for Scorodonin (the 4th molecule), BR-SAScore shows a strong correlation with both literature reports and synthesis planning results. The comparison with other methods in Figure S7 reveals that RAScore and CLScore tend to overestimate the synthetic accessibility of HS molecules, while SYBA underestimates the accessibility of ES molecules. GASA exhibits a similar prediction trend to DeepSA.

The simplicity of R^2^fragmentScore in BR-SAScore calculation, based solely on the scores of chemical fragments existing within the molecule, facilitates straightforward score interpretation. Negative R^2^fragmentScores highlight fragments that are infrequently observed in the reaction center of the reaction database or within molecules from accessible building blocks. Figure 4c–f highlight the contribution of negative R^2^fragmentScores from each atom (center of chemical fragment) for four tested molecules successfully solved by Retro*, Gabapenin, Sedridine, Scorodonin, and Pinnatolide, representing varying levels of synthetic difficulty planned by Retro*. For instance, Gabapenin (Fig. 4c), lacking hard-to-synthesize fragments, exhibits a low BR-SAScore at 2.5. Conversely, the presence of difficult-to-synthesize C–C bonds in molecules like Sedridine (Fig. 4d) and Pinnatolide (Fig. 4f) contribute to medium BR-SAScore at 5.03 and 5.21, respectively. The conjugated allene structure in Scorodonin (Fig. 4c) is recognized as challenging to synthesize, resulting in a high BR-SAScore at 8.06.

**Fig. 5:**
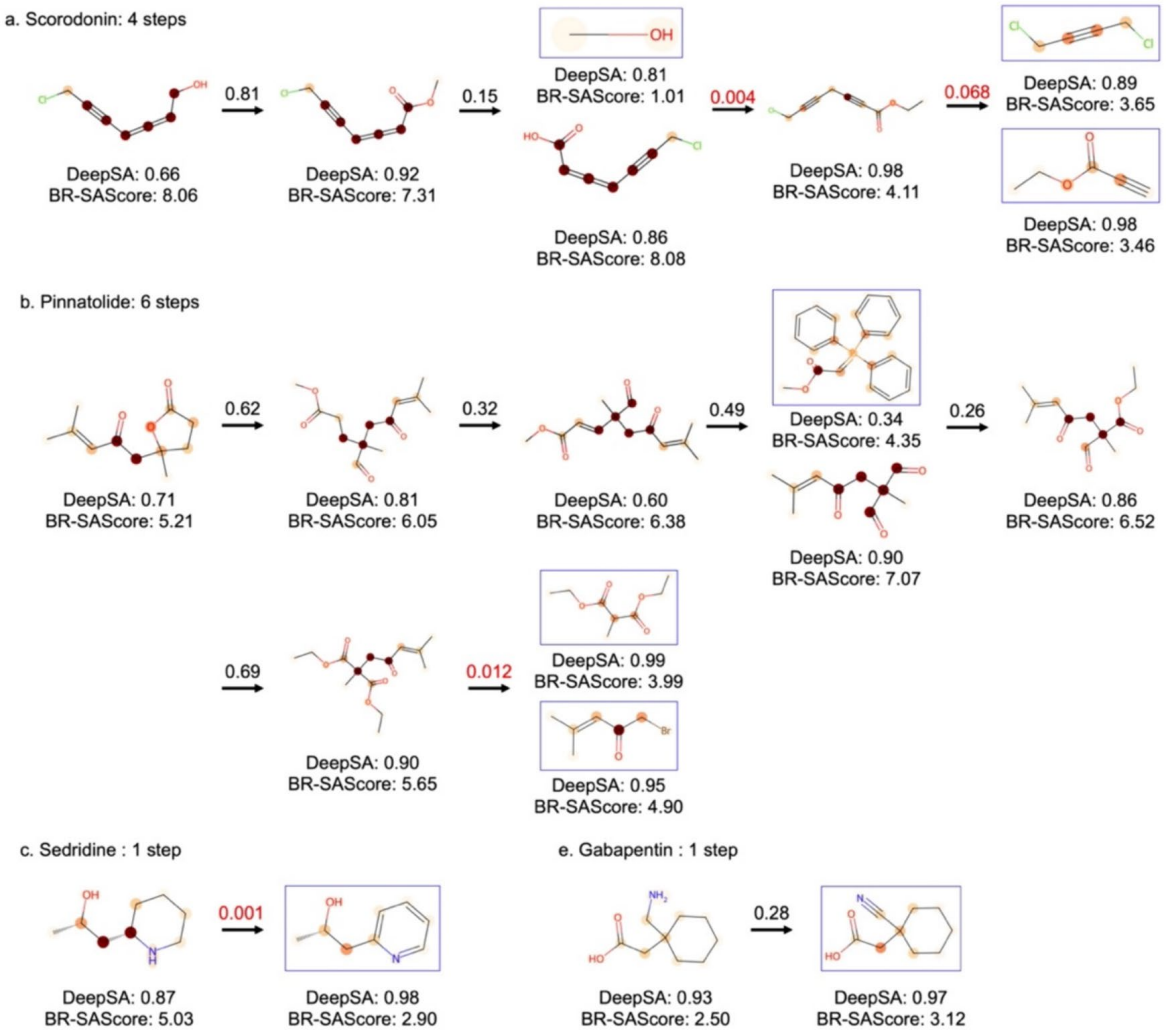
The DeepSA score and BR-SAScore for four molecules, (**a**) Scorodnin, (**b**) Pinnatolide, (**c**) Sedridine, and Gabapentin, and their precursors in the synthesis routes predicted by Retro*. Accessible building blocks are displayed in blue boxes. The values above the arrow are the prediction scores of the single-step prediction model of Retro*, where the prediction scores lower than 0.1 are highlighted in red color

We further analyze the BR-SAScore of each molecule, highlighted by its atom contributions, during the synthesis planning process of the four molecules shown in Fig. 4c–f, predicted by Retro*, and compared with DeepSA as depicted in Fig. 5. For Scorodonin (Fig. 5a), the BR-SAScore of the molecules in the first three retrosynthesis steps is very low due to the presence of conjugated allene structure. Subsequently, the BR-SAScore experiences a notable decrease from 8.08 to 4.11 after the third reaction step, further drops to 3.65 and 3.46 after the last retrosynthesis step due to the absence of conjugated allene structure in the molecule's structure. However, the third retrosynthesis step is predicted with a very low retrosynthesis score (0.004), indicating an unclear predicted reaction mechanism. Regarding Pinnatolide (Fig. 5b), the BR-SAScore of the molecules following the initial five retrosynthesis steps remains higher than the target molecule due to the alkane chain formation between three carbonyl groups post the retrosynthetic ring-forming reaction. Notably, the BR-SAScores of the molecules surge below 5 following an S_N_2 reaction attacking the acetic acid, with a retrosynthesis score of 0.012. For Sedridine (Fig. 5c) and Gabapentin (Fig. 5d), Retro* predicts the synthesis planning completion in one step. The retrosynthesis score for the formation of the asymmetric C–C bond through hydrogenation reaction for Sedridine synthesis (0.001) stands notably lower compared to the nitrile reduction for Gabapentin synthesis (0.28).

Overall, the BR-SAScores of the molecules align well with the confidence levels of synthesis planning program. Specifically, the BR-SAScore tends to increase only after a low-score retrosynthesis prediction, indicating the same challenge for synthesis planning software to resolve the hard-to-synthesize chemical fragments. In contrast, the DeepSA scores for the four target molecules and most precursors are consistently high, surpassing 0.5. Notably, the only precursor showing low DeepSA score (0.34) is the Wittig reagent used at the third retrosynthesis step of Pinnatolode available in the building blocks, and the reason of the low DeepSA score is not interpretable. This discrepancy underscores the significance of distinguishing fragments in building blocks and fragments derived from synthesis (reactions) when scoring synthetic accessibility.

Note that predicted synthetic accessibility from GASA [25] is also explainable by visualizing the predicted attention of each atom in the molecule. Therefore, we depicted a similar figure to Fig. 5 in Figure S8 to analyze the explainability of synthetic accessibility prediction using GASA. However, we did not find any straightforward correlation between the synthetic accessibility of molecules and the atoms highlighted by GASA attention. The scores predicted by all 7 methods are available in Figure S9. Both SYBA and SAScore show a positive correlation with the Retro* prediction in terms of score changes after the low-score route prediction, while the other methods do not exhibit changes after the low-score route prediction.

## Conclusion

In this study, we introduce BR-SAScore, a building block and reaction-aware adaptation of SAScore, which considers reaction-driven fragments and building-block-accessible fragments relevant to the synthesis planning software. Our experiments demonstrated that BR-SAScore provides a better prediction correlation with the synthesis planning software compared to the original SAScore and other existing methods, including deep-learning approaches, all within a short calculation time (~ 0.42 ms per molecule). From a chemical perspective, the superior performance of BR-SAScore can be attributed to its consideration of finite reaction knowledge and the available building blocks within the synthesis planning software. Since we are using the reaction data (USPTO) and commercially available building blocks (eMolecules) used in Retro* to score reaction-driven and building block fragments, one can view our method as a simplified but much faster model to mimic Retro* to estimate synthesizability (but without actual pathways). We note that our scoring method is applicable to any synthesis planning program, and if more advanced retrosynthesis planning models are developed in the future, our scoring pipeline can still be used the same way as for Retro* but using different reaction and building block data.

In addition, the chemically intuitive design of BR- SAScore facilitates straightforward interpretation of the calculated scores by highlighting the contributions from essential chemical fragments. By examining changes in BR- SAScore for precursor molecules in predicted synthesis routes, we illustrate how these highlighted chemical fragments are important in understanding the reasons for low synthetic accessibility from a chemical perspective. Given its adaptability to any reaction dataset and knowledge of building blocks, we anticipate that BR- SAScore will significantly aid in the practical estimation of synthetic accessibility for virtually designed chemicals in the future.

### Supplementary Information

Supplementary Material 1.

## Data Availability

All the data sets and source code are publicly available through GitHub (https://github.com/snu-micc/BR-SAScore).
