# Supplementary material for: Estimating the synthetic accessibility of molecules with building block and reaction-aware SAScore
Source: J Cheminform. 2024 Jul 23;16:83. doi: 10.1186/s13321-024-00879-0 (PMC11267797; doi:10.1186/s13321-024-00879-0)
Supplement: Supplementary file 1 — Supplementary Material 1. [file 13321_2024_879_MOESM1_ESM.docx]

Supplementary Information

Estimating the Synthetic Accessibility of Molecules with Building block and Reaction-aware SAScore

Shuan Chen^1^, Yousung Jung^1,2*^

1. Department of Chemical and Biological Engineering, Seoul National University, 1 Gwanak-ro, Gwanak-gu, Seoul, 08826, South Korea
2. Institute of Chemical Processes, Seoul National University, 1 Gwanak-ro, Gwanak-gu, Seoul, 08826, South Korea
3. Institute of Engineering Research, Seoul National University, 1 Gwanak-ro, Gwanak-gu, Seoul, 08826,

South Korea

*Correspondence: [yousung.jung@snu.ac.kr](mailto:yousung.jung@snu.ac.kr)

# S1. Hyperparameters of Retro*

Table S1. The hyperparameters of Retro*[1] implemented in this paper for synthetic accessibility labeling.

| **Hyperparameter** | **Value** |
| --- | --- |
| Use GPU | False |
| Use value function | True |
| Maximum iteration | 100 |
| Expansion topk | 50 |

# S2. Distribution of the number of atoms and number of chiral centers


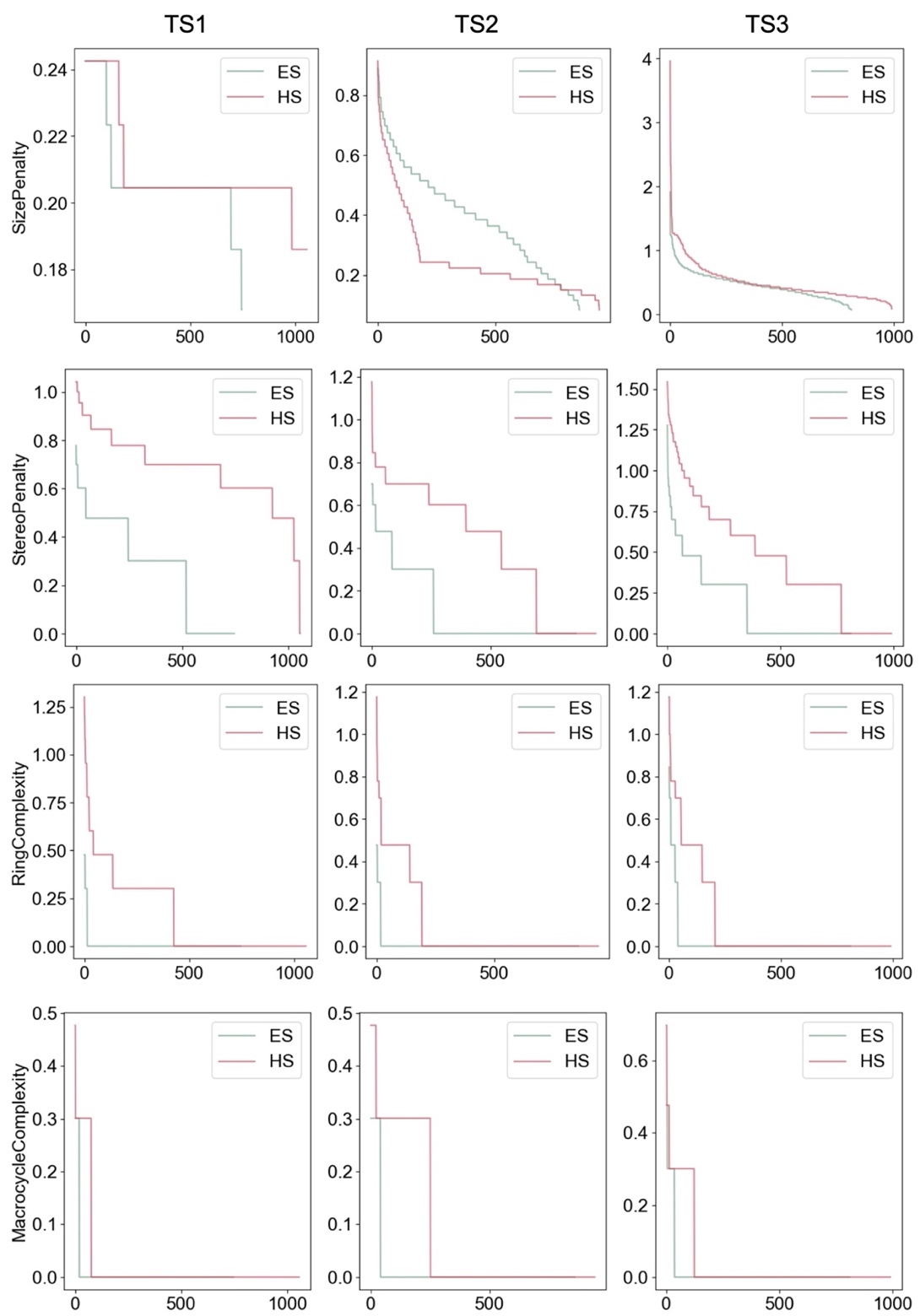


Figure S1. The distribution of the SizePenalty, StereoPenalty, RingComplexity, MacrocycleComplexity of ES and HS molecules in the three test sets.

# S3. Top-10 RFrags and BFrags with the highest score


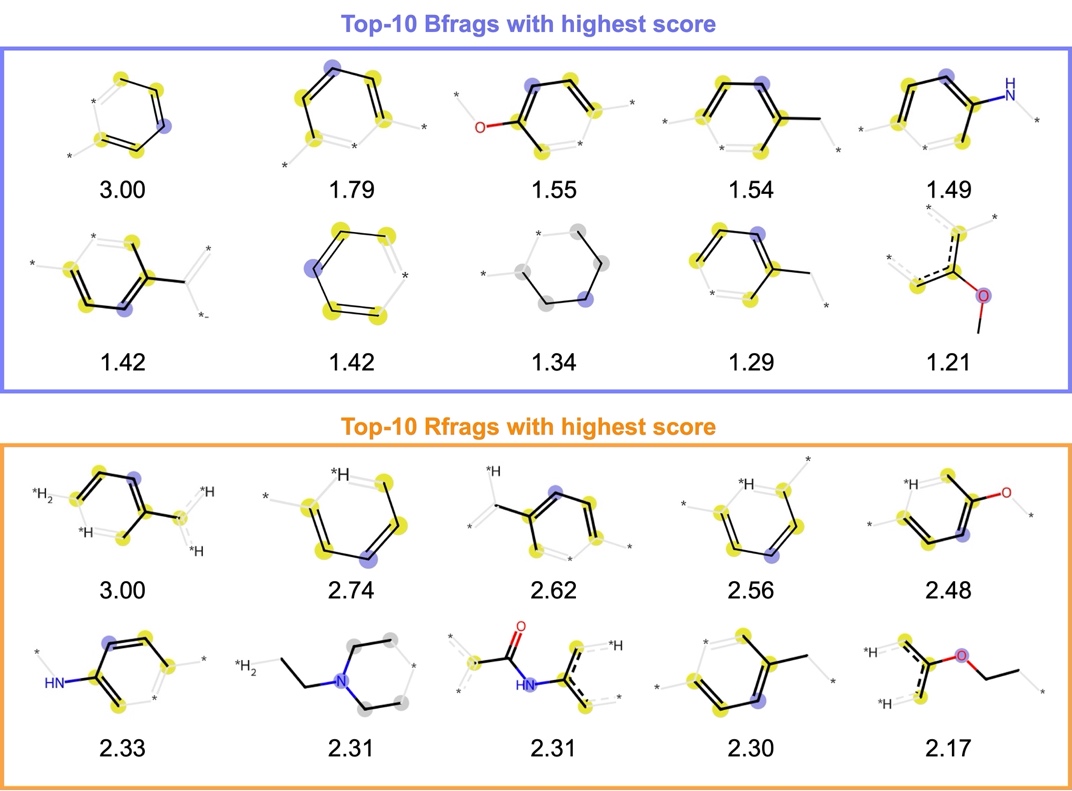


Figure S2. The ECFP4[2] of the top-10 BFrags and RFrags with the highest score derived from the eMolecules building blocks (https://downloads.emolecules.com/free) and the USPTO reaction dataset[3].

# S4. Raw prediction scores


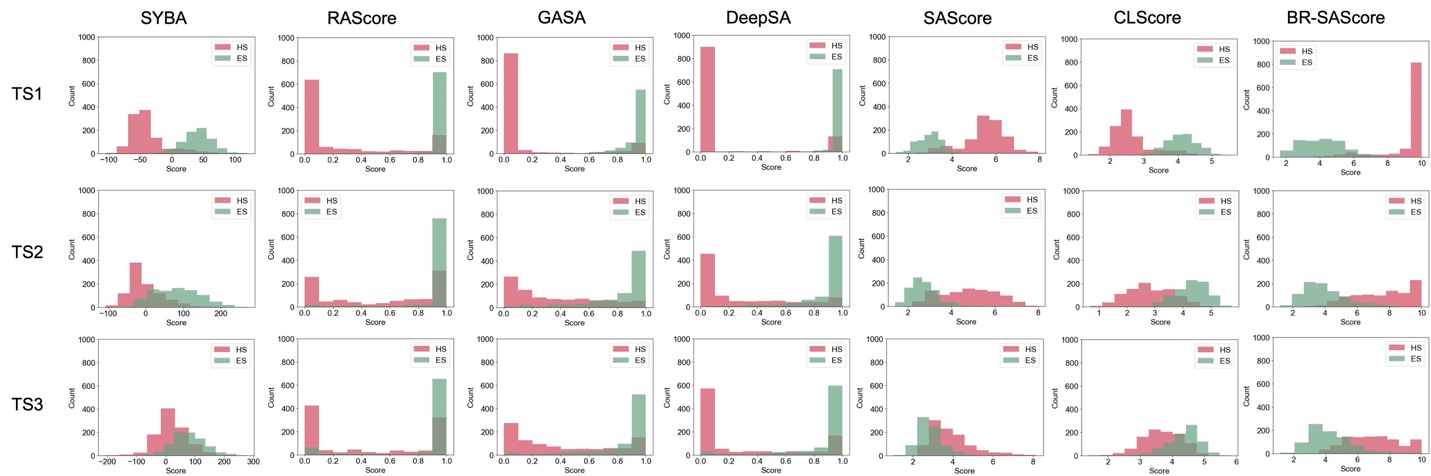


Figure S3. The raw prediction scores of BR-SAScore compared with the 6 existing methods on the three test sets.

# S5. Ablation study


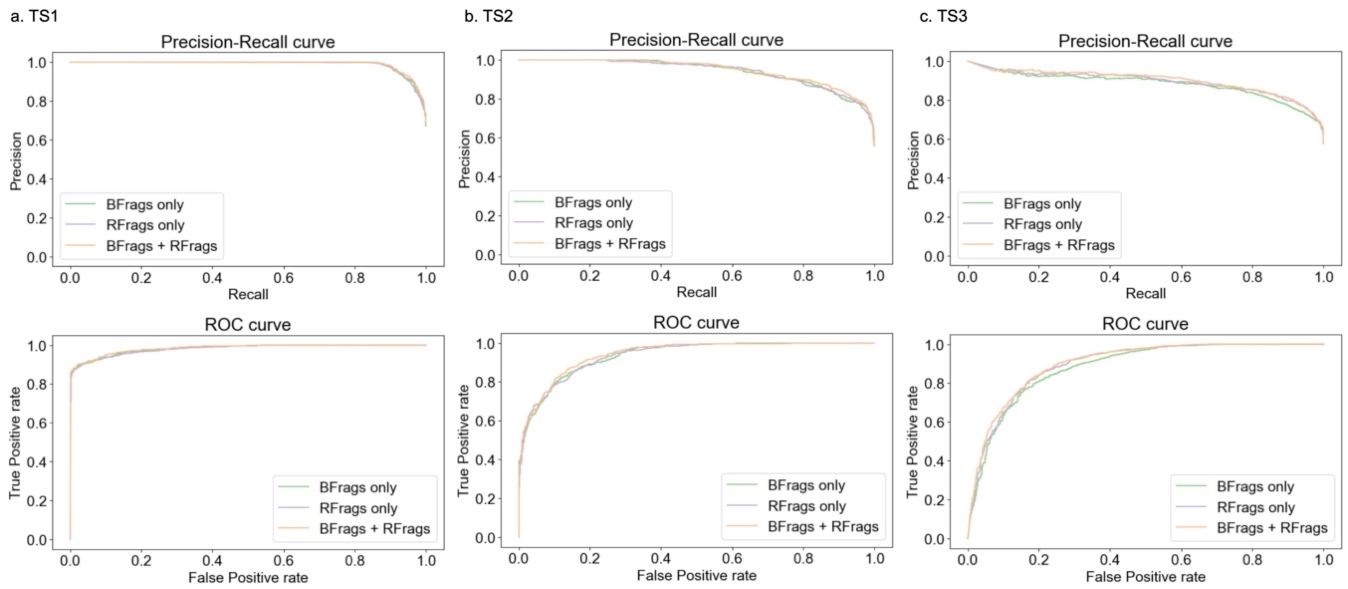


Figure S4. precision-recall and ROC curves of BR-SAScore using only RFrags or RFrags calculated on the three test sets.

Table S2. Ablation study of BR-SAScore using only BFrags or RFrags. The best values are highlighted in font bond and the second-best values are underlined.

| Variant | PR-AUC | | | ROC-AUC | | |
| --- | --- | --- | --- | --- | --- | --- |
|  | TS1 | TS2 | TS3 | TS1 | TS2 | TS3 |
| BFrags only | 0.989 | 0.941 | 0.883 | 0.982 | 0.935 | 0.883 |
| RFrags only | 0.987 | 0.940 | 0.894 | 0.980 | 0.934 | 0.895 |
| BFrags + RFrags | **0.990** | **0.947** | **0.900** | **0.984** | **0.942** | **0.990** |


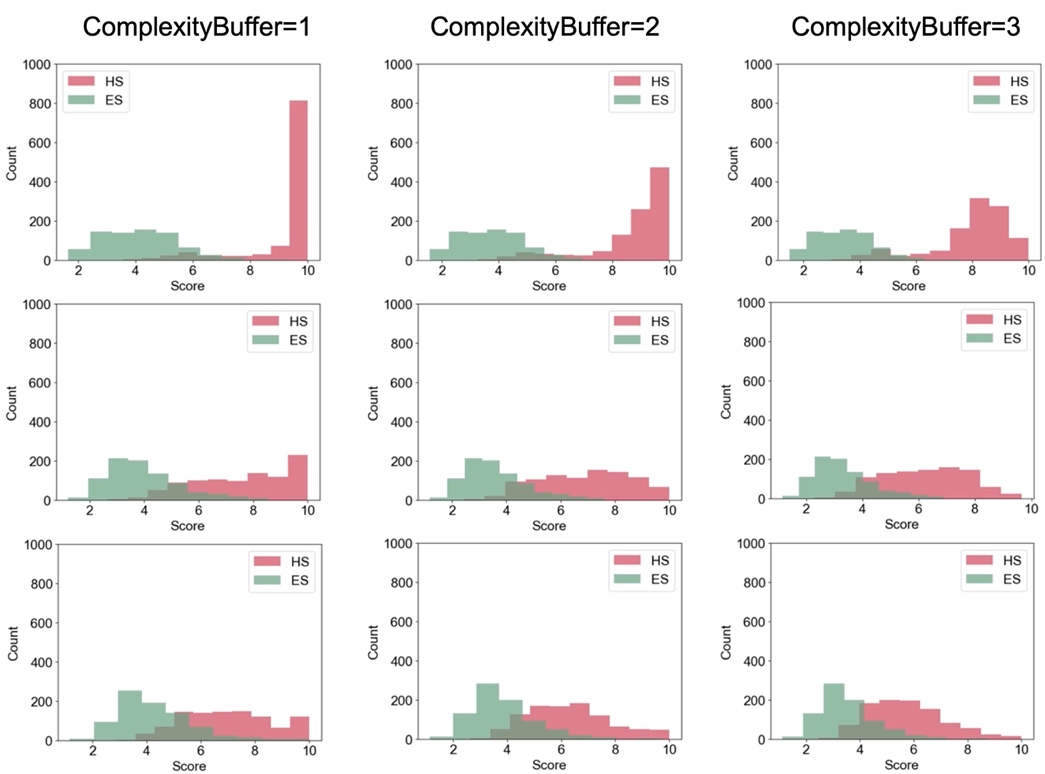


Figure S5. The raw predictions of BR-SAScore with complexity buffer 1, 2, and 3 calculated on the three test sets.


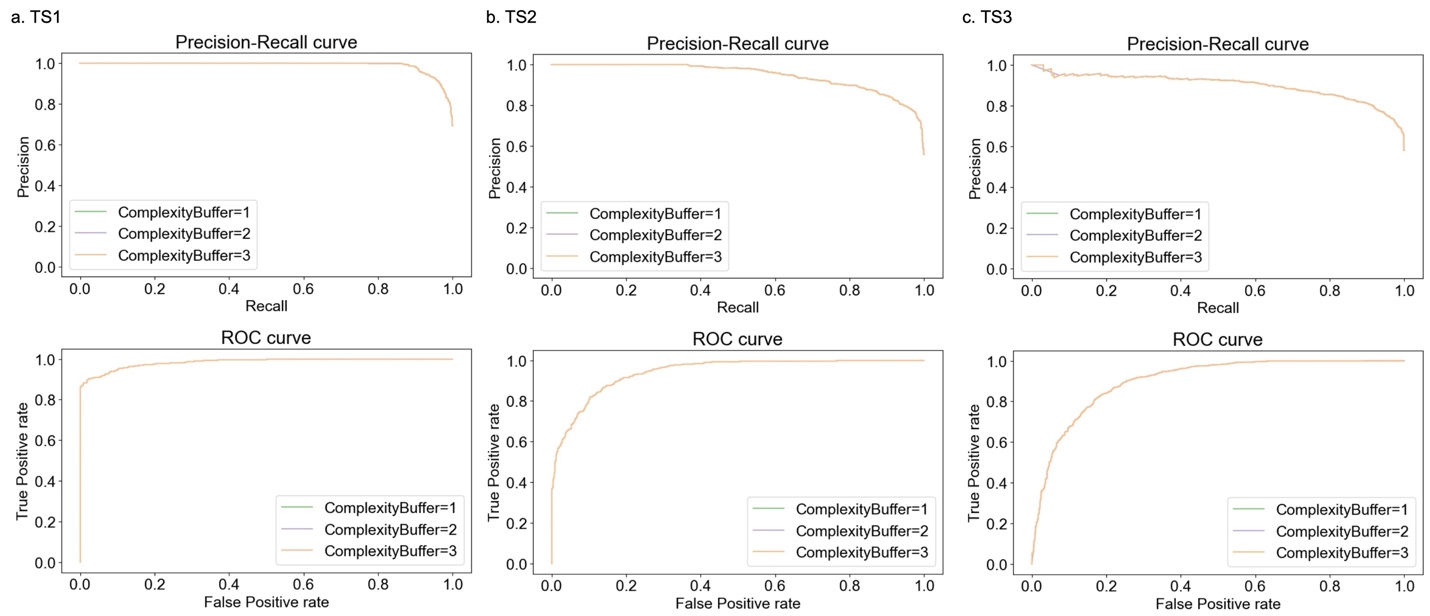


Figure S6. The precision-recall and ROC curves of BR-SAScore with complexity buffer 1, 2, and 3 calculated on the three test sets.

# S6. List of complex molecules


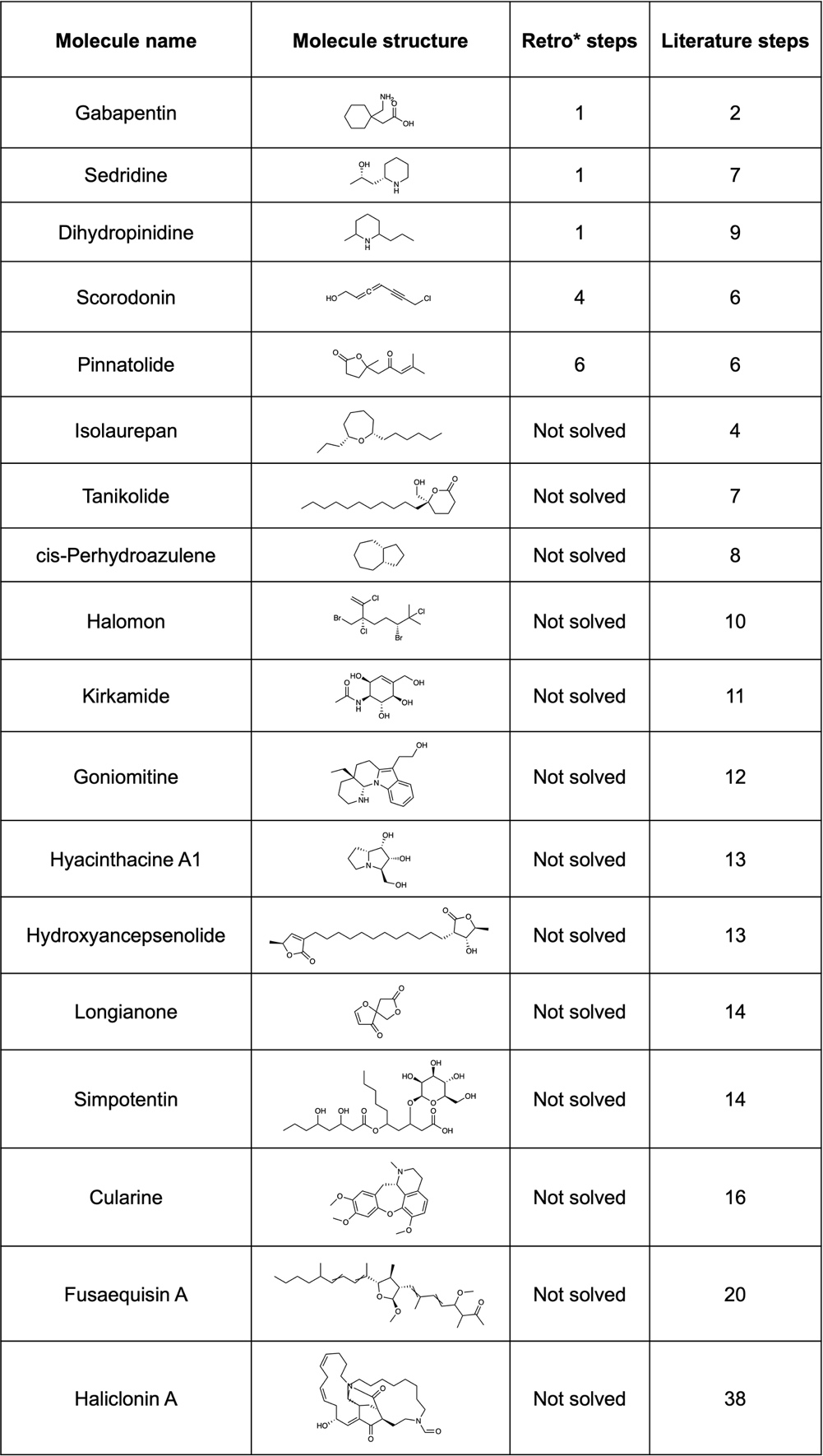


Table S3. The complex molecules collected by Wang et al.[4] and the number of synthesis steps planned by Retro*[1] and reported in the literatures.

# S7. Predictions on complex molecules


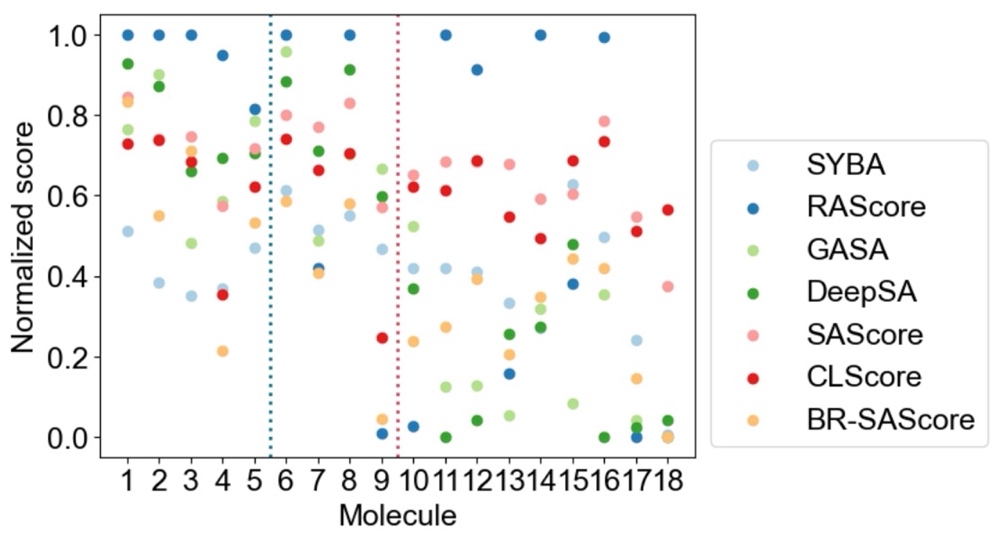


Figure S7. The normalized synthesis accessibility scores predicted by all the 7 methods on 18 complex molecules collected by Wang et al.[4]

# S8. Predicted scores on synthesis routes


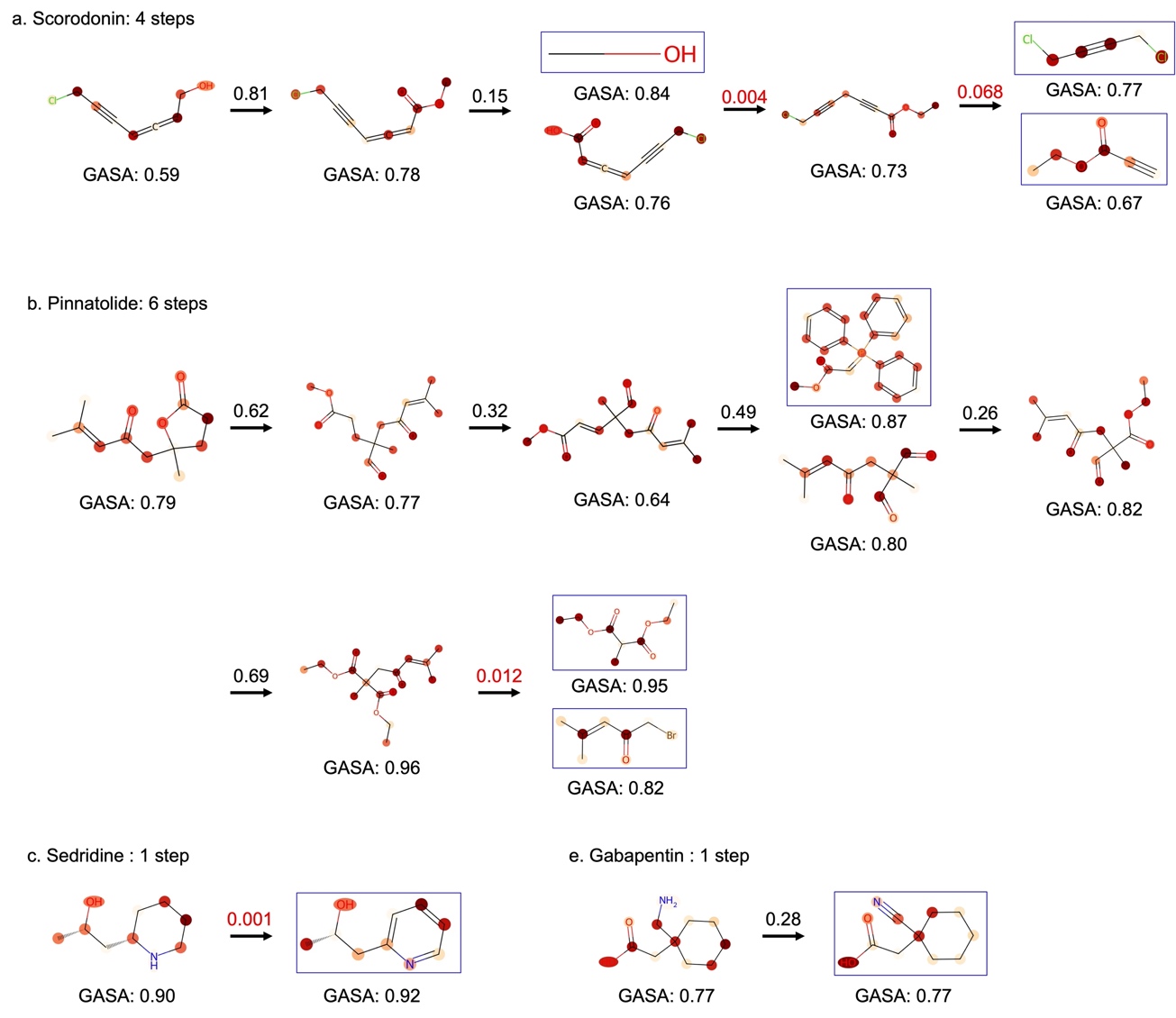


Figure S8. The GASA score[5] for four molecules, (a) Scorodnin, (b) Pinnatolide, (c) Sedridine, and Gabapentin, and their precursors in the synthesis routes predicted by Retro*. Accessible building blocks are displayed in blue boxes. The values above the arrow are the prediction scores of the single-step prediction model of Retro*, where the prediction scores lower than 0.1 are highlighted in red color.


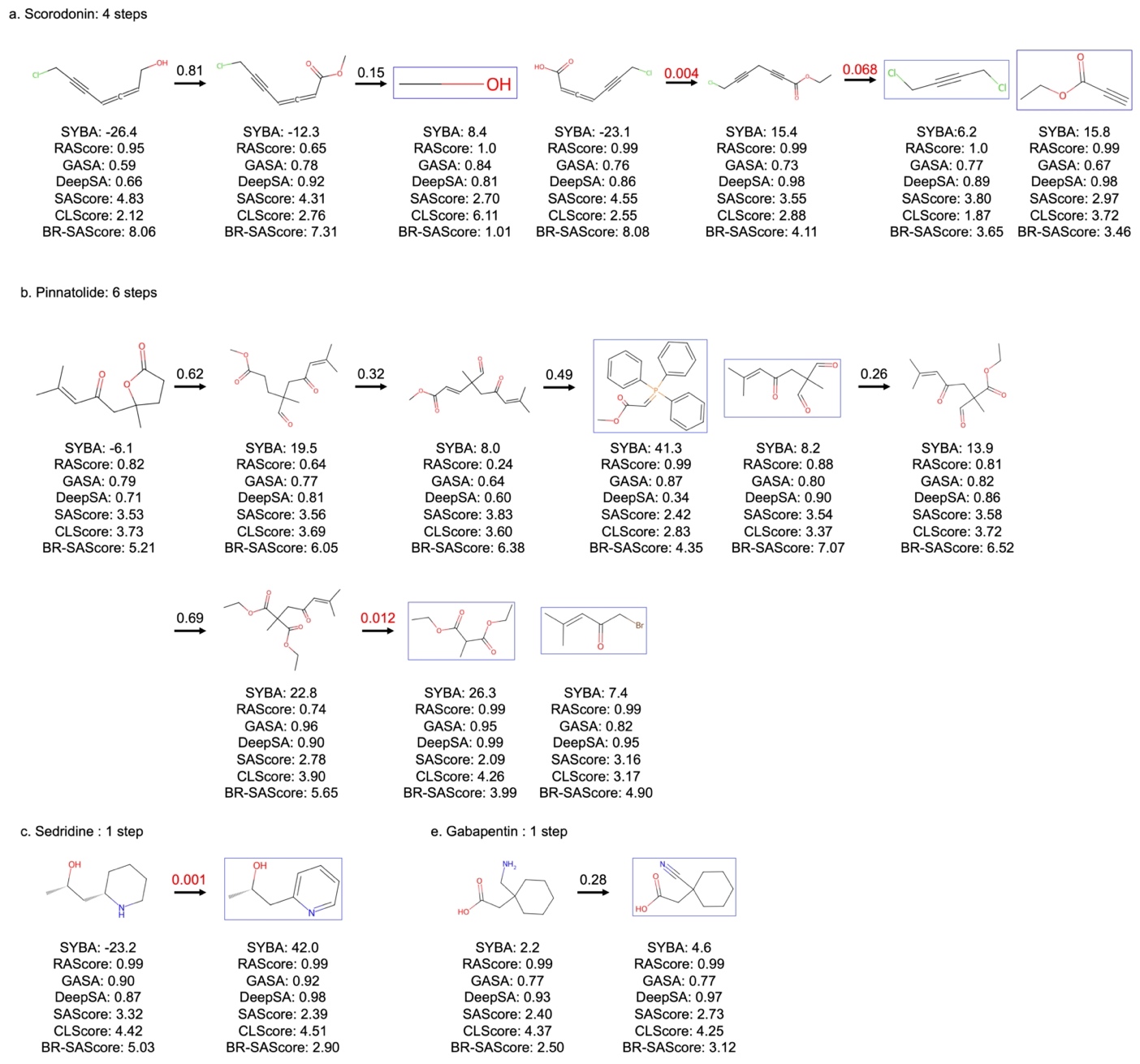


Figure S9. The scores predicted by all 7 methods on four molecules, (a) Scorodnin, (b) Pinnatolide, (c) Sedridine, and Gabapentin, and their precursors in the synthesis routes predicted by Retro*. Accessible building blocks are displayed in blue boxes. The values above the arrow are the prediction scores of the single-step prediction model of Retro*, where the prediction scores lower than 0.1 are highlighted in red color.

# References

1. Chen B, Li C, Dai H, Song L (2020) Retro*: Learning Retrosynthetic Planning with Neural Guided A* Search. In: Proceedings of the 37th International Conference on Machine Learning. PMLR, pp 1608–1616

2. Rogers D, Hahn M (2010) Extended-Connectivity Fingerprints. J Chem Inf Model 50:742–754. https://doi.org/10.1021/ci100050t

3. Lowe DM (2012) Extraction of chemical structures and reactions from the literature. Thesis, University of Cambridge

4. Wang S, Wang L, Li F, Bai F (2023) DeepSA: a deep-learning driven predictor of compound synthesis accessibility. J Cheminformatics 15:103. https://doi.org/10.1186/s13321-023-00771-3

5. Yu J, Wang J, Zhao H, et al (2022) Organic Compound Synthetic Accessibility Prediction Based on the Graph Attention Mechanism. J Chem Inf Model 62:2973–2986. https://doi.org/10.1021/acs.jcim.2c00038
